# Relationships between frequency of family meals, BMI and nutritional aspects of the home food environment among New Zealand adolescents

**DOI:** 10.1186/1479-5868-5-50

**Published:** 2008-10-23

**Authors:** Jennifer Utter, Robert Scragg, David Schaaf, Cliona Ni Mhurchu

**Affiliations:** 1 Epidemiology and Biostatistics, School of Population Health, University of Auckland, Auckland, New Zealand; 2 Pacific Health, School of Population Health, University of Auckland, Auckland, New Zealand; 3 Clinical Trials Research Unit, School of Population Health, University of Auckland, Auckland, New Zealand

## Abstract

**Background:**

Previous research has documented the positive effects of family meals on the dietary quality of adolescents. The objective of the current study is to examine associations between frequency of family meals and body mass index (BMI), other aspects of the home food environment, and related nutrition behaviors.

**Methods:**

Data were collected during baseline measurements of the Pacific Obesity Prevention In Communities study. In total, 3245 ethnically diverse students completed a questionnaire about their nutrition behaviors and were weighed and measured for height.

**Results:**

In total, 42% of adolescents ate a family meal on all of the previous five school nights. Frequency of family meals was modestly associated with BMI in bivariate analysis (p = 0.045), but lost significance when demographic characteristics were included in the model. Frequency of family meals was associated with many positive aspects of home food environment and positive nutrition behaviors, including parental support for healthy eating, limits on television use, having fruit available at home, consuming five fruits and vegetables a day, eating breakfast, and bringing lunch from home. Surprisingly, no relationships were observed between frequency of family meals and accessibility and consumption of many high fat/high sugar foods.

**Conclusion:**

Our findings suggest that the positive effect of family meals may reflect an overall positive home food environment. Families who have meals together have more healthful foods available at home and support their child in eating healthfully. There were no relationships between family meals and high fat/high sugar foods; this suggest that while families may prioritize eating together, messages about limiting the availability and consumption of these snack foods are not getting through.

## Background

The family and home environment plays an important role in adolescent nutrition as it has been estimated that adolescents consume about 60% of their daily energy from foods sourced at home [1]. Accessibility of healthy and unhealthy foods at home, parental modelling of healthy eating, family eating patterns and work demands all influence the eating practices of children and families [2,3]. Family meals are one aspect of the home environment associated with the overall well-being of adolescents. Frequency of family meals has been inversely associated with substance use [4] and positively associated with mental health [4], school achievement [4], as well as dietary quality among adolescents. Adolescents who eat meals with their families eat more fruits and vegetables [5-7], drink fewer soft drinks [5,6], are more likely to eat breakfast [7], and have better nutrition profiles, especially for calcium [6] and saturated fat [5]. Furthermore, regular consumption of family meals during adolescence is associated with higher consumption of regular meals, vegetables and some nutrients during young adulthood [8].

Given the nutritional benefits of eating meals as a family, it is logical to hypothesize that frequent consumption of family meals would be associated with reduced prevalence of overweight/obesity. Few cross-sectional epidemiological studies have examined the relationships between family meals and body weights [9,10]; these studies are limited by the use of self-reported measures of height and weight and the findings of these studies are inconsistent. At least two longitudinal studies have examined the effects of family meals on body weights over time. One study of young adolescents reported that eating meals as a family was not a significant predictor of becoming overweight (determined with self-reported height and weight) one year later [10]. Another study of younger children reported that those eating the fewest family meals at kindergarten were significantly more likely to become overweight by the third grade [11]. To date, there is a paucity of research examining the relationship between family meals and body mass index (BMI) among a large population of adolescents using objective measures of BMI.

Existing research is also limited in its ability to determine what accounts for the positive relationships between family meals and adolescent nutrition, but increased availability of healthy foods at home, discussion about nutrition at family meals, and/or modelling of healthy eating are all possible mechanisms [12]. Therefore, the objectives of the current study are: a.) to examine the relationship between consumption of family meals and objectively measured BMI among a large, diverse population of adolescents and b.) to examine the associations between frequency of eating family meals and other aspects of the home food environment and nutrition behaviors that may influence adolescent nutrition.

## Methods

Data were collected during the baseline measurements for the Pacific Obesity Prevention In Communities (OPIC) study: a muti-site intervention aiming to reduce overweight/obesity among predominately Pacific adolescents. Data for the current study were collected at the New Zealand site; study participants were drawn from seven high schools in a geographically defined, economically disadvantaged area during the 2005 school year. All seven schools were deciles one or two. School deciles reflect the socioeconomic position of the attending students and range from one to 10; decile-one represents most deprived while decile-ten represents the least deprived. One school of the original seven was excluded from this analysis due to poor student response rate (25%).

All students attending the school during the days of data collection were invited to participate. From the remaining six schools, 3245 students agreed to participate (response rate 62%). Final analyses were conducted with the 3119 students who had complete survey data about nutrition and physical activity patterns and physical measurements. Parents of students under age 16 and students aged 16 years and older consented to participation. The University of Auckland Human Participants Ethics Committee granted ethical approval for the study.

### Measures

Height and weight measurements were taken by trained research staff using standardized protocols. Students wore light clothing and no shoes. Students' weights were measured to the nearest 0.1 kilogram using a digital scale. Students' heights were measured to the nearest 0.1 centimeter using a free-standing portable stadiometer. Body mass index (BMI) was calculated as weight (kilograms) divided by height (meters) squared.

All remaining measures were assessed by self-report using a handheld computer. The survey items were pilot tested for comprehension with four school classes, one class at each school level. Frequency of *family meals *was assessed with the question, "In the last 5 school days, how many times did all or most of your family living in your house eat an evening meal together?" Response options ranged from 0 to 5 days and were collapsed into "none," "1 to 2 days," "3 to 4 days," and "everyday" for analysis.

*Mother/father support for healthy eating *were assessed with two questions asking how much does your mother (or female caregiver)/father (or male caregiver) encourage you to eat healthy foods. Students could respond "a lot," "some," "a little," or "not at all." For analysis, response options for mother/father support for healthy eating were dichotomized into "a lot" or "some, a little, or not at all. "*Parental limits on television use *was assessed by asking students if their parents limited the amount of TV, videos and DVDs they were allowed to watch during the school week. Response options included "no limits," "yes, but not very strict limits," or "yes, strict limits." Response options were dichotomized for analysis into "yes, strict limits or yes, but not very strict limits" or "no." *Home availability of fruit, chips, chocolates, and soft drinks *were assessed by four separate questions each with the responses "everyday," "most days," "some days," or "hardly ever". Home availability of fruit was dichotomized at "everyday" and "most days, some days, or hardly ever." Home availability of chips, chocolates and soft drinks were dichotomized at "everyday or most days" or "some days or hardly ever."

Students were asked about their frequency *breakfast consumption *and *soft drink consumption *over the last five school days. These two items were measured in relation to the previous five school days because they were directly relevant to key intervention strategies of the study. For all other nutrition behaviours students were asked to report their usual consumption. It is generally easier for subjects to describe their usual consumption of foods, rather than what was eaten at a specific time in the past [13]. This concept refers to generic memory (rather than episodic) and has been frequently used by epidemiologists investigating food-borne illnesses [13]. Two separate items assessed usual daily *fruit and vegetable consumption*. Responses were summed and dichotomized to reflect national recommendations for fruit and vegetable consumption (5 or more per day) [14]. *Bringing lunch from home *was assessed with one question asking where students usually got their lunch from. Students could choose from home, school canteen, shops (takeaway food outlet or convenience stores), friends, or that they did not eat lunch. Options were dichotomized into "home" or "school, shops, friends or no lunch" for analysis. Usual *fast food/takeaway food consumption *was assessed with a single question with five response options ranging "most days" to "once a month or less." Responses were dichotomized at once a week or more. Frequency of eating fried food, chocolates, sweets or ice cream, and fruit as *after school snacks *were assessed with four separate items. Responses included "everyday or almost everyday," "most days," "some days," and "hardly ever or never" and were dichotomised at "most or some days." Student's *ethnicity*, *age *and *gender *were each assessed by self-report.

### Analysis

All analyses were conducted using SAS (version 9.1, Cary, NC) using the SURVEY procedures to adjust for the clustered sample design. Demographic characteristics of students by frequency of family meals were generated by cross-tabulations using chi-square tests for statistical significance. The association between consuming family meals and BMI was generated with regression models, controlling for age and gender. The associations between frequency of family meals and other aspects of the home food environment were generated using logistic regression models, controlling for age and gender. Additional measures of socioeconomic status were not measured in this survey because all of the participating schools were deciles 1 or 2.

## Results

The final study sample comprised slightly more females (52%) than males (48%) and had a mean age of 14.8 years. Students in the final study sample identified their ethnicity primarily as Pacific (63%), Maori (19%), Asian/other (11%) and European (8%). The sample had a high prevalence of overweight/obesity; 58% of the study sample were classified as overweight or obese. Detailed information on the weight status of the sample by age, gender and ethnicity have been previously reported [15].

In total, 42% of students had a meal with their family on all of the past five school nights (Table 1). Age was significantly associated with frequency of family meals (p < 0.001) such that older adolescents were less likely to eat family meals everyday than younger adolescents. Males were slightly more likely to eat family meals everyday than females (p = 0.049). Ethnicity was not associated with frequency family meals.

**Table 1:** Demographic characteristics of adolescents by frequency of consuming a meal with their family during the previous school week

**Frequency of family meals**		**None**		**1–2 days**		**3–4 days**		**Everyday**		
	**n**	**%**^1^	**SE**	**%**	**SE**	**%**	**SE**	**%**	**SE**	**p-value**

**Total**	3119	9.6	0.6	17.9	0.7	30.2	0.9	42.4	1.1	
										
**Gender**										
Male	1496	8.0	0.7	17.0	1.0	31.8	1.2	43.2	1.4	
Female	1623	11.1	0.9	18.7	1.0	28.7	1.1	41.6	1.5	0.049
										
**Ethnicity**										
Pacific Island	1949	8.6	0.7	19.1	0.9	31.2	1.0	41.0	1.3	
Maori	603	10.3	1.2	16.7	1.3	30.0	2.3	43.0	2.1	
Asian	335	11.3	1.6	14.6	1.8	27.2	2.4	46.9	2.7	
European	232	13.8	2.0	15.1	2.6	25.9	3.2	45.3	3.2	0.55
										
**Age**										
≥ 13	733	8.6	1.1	17.2	1.6	28.1	1.8	46.1	1.9	
14	780	8.8	1.2	16.2	1.2	30.9	1.7	44.1	2.0	
15	600	10.5	1.3	14.5	1.5	29.7	2.0	45.3	2.2	
16	538	9.3	1.1	22.3	1.6	31.8	2.2	36.6	2.3	
≥ 17	468	11.8	1.7	20.9	1.9	31.0	2.1	36.3	2.1	< 0.001

^1^Standard Error for the percent

The relationship between frequency of family meals and BMI was significant in the bivariate analysis (p = 0.045), but relationship was modest as the confidence intervals were over-lapping (Table 2). Students eating meals with their families on all of the previous five school nights had a lower mean BMI than those who did not eat any meals with their families. When age and gender were treated as confounders in the model, the relationship was no longer significant.

**Table 2:** Relationship between frequency of eating family meals in past school week and BMI

	**Mean BMI**	**CI**^1^	**Bivariate p-value**	**β**^2^	**SE**^3^	**Multivariate p-value**^4^
Frequency of family meals						
None	26.13	(25.4,26.8)		0.0	0.0	
1–2 days	26.67	(26.2,27.2)		0.58	0.4	
3–4 days	25.91	(25.5,26.3)		-0.05	0.4	
Everyday	25.88	(25.5,26.2)	0.045	0.01	0.4	0.22

^1 ^95% Confidence interval for the mean

^2 ^β coefficient for multivariate model examining age and gender simultaneously

^3 ^Standard error for β coefficient

^4 ^P-value for multivariate model examining age and gender simultaneously

Frequency of family meals was positively associated with many of the more healthful aspects of the home food environment (Table 3). Adolescents that reported having family meals everyday were significantly more likely to perceive a lot of maternal (p < 0.001) and paternal (p < 0.001) support for healthy eating, have limits on their television use (p < 0.001), and have fruit available in their home every day (p < 0.001). Frequency of family meals was also positively associated with eating five fruits and vegetables a day (p < 0.001), eating fruit as an afternoon snack (p < 0.001), bringing lunch from home (p < 0.001), and eating breakfast at home before school (p < 0.001). Furthermore, in many cases, the strength of the associations increased with the frequency of eating family meals.

**Table 3:** Odds ratios (OR) and 95% Confidence Intervals (CI) describing the relationships between frequency of family meals and aspects of the home food environment and nutrition behaviors

**Frequency of family meals**		**None**	**1–2 days**	**3–4 days**	**Everyday**	p-value^1^
**Support and Boundaries**						
A lot of maternal support for healthy eating	%	49.7	55.3	58.6	67.3	
	OR	1.0	1.28	1.46	2.09	
	CI	.	0.9–1.7	1.1–1.9	1.6–2.7	< 0.001
						
A lot of paternal support for healthy eathing	%	41.2	44.5	44.6	54.4	
	OR	1.0	1.16	1.15	1.69	
	CI	.	0.9–1.5	0.9–1.5	1.3–2.2	< 0.001
						
Parental limits on TV use	%	42.3	54.0	56.4	60.2	
	OR	1.0	1.61	1.75	2.00	
	CI	.	1.2–2.1	1.4–2.2	1.6–2.5	< 0.001
						
**Food Accessibility at home**						
Fruit available everyday	%	54.7	50.3	55.3	63.1	
	OR	1.0	0.85	1.04	1.44	
	CI	.	0.7–1.1	0.8–1.4	1.1–1.8	< 0.001
						
Chips available most days	%	41.3	43.1	42.8	40.3	
	OR	1.0	1.07	1.06	0.96	
	CI	.	0.8–1.4	0.8–1.4	0.7–1.2	0.58
						
Chocolates available most days	%	24.7	25.3	24.9	23.8	
	OR	1.0	1.04	1.01	0.95	
	CI	.	0.8–1.4	0.8–1.3	0.7–1.3	0.87
						
Soft drinks available most days	%	35.0	37.3	37.6	34.1	
	OR	1.0	1.09	1.10	0.95	
	CI	.	0.8–1.5	0.8–1.5	0.7–1.2	0.43
						
**Positive Nutrition Behaviors**						
5 or more fruits and vegetables a day	%	33.7	37.7	39.3	47.8	
	OR	1.0	1.19	1.246	1.76	
	CI	.	0.9–1.6	1.0–1.6	1.4–2.3	< 0.001
						
Fruit for afternoon snack, most days	%	33.7	38.1	40.7	47.5	
	OR	1.0	1.23	1.37	1.76	
	CI	.	0.9–1.7	1.1–1.9	1.3–2.3	< 0.001
						
Bring some of their school food from home	%	63.7	64.3	71.9	78.7	
	OR	1.0	1.01	1.40	1.99	
	CI	.	0.8–1.4	1.1–1.9	1.5–2.7	< 0.001
						
Eat breakfast at home before school, everyday	%	18.3	13.6	22.0	31.6	
	OR	1.0	0.70	1.22	1.97	
	CI	.	0.5–1.1	0.8–1.8	1.4–2.8	< 0.001
						
**Less Healthy Nutrition Behaviors**						
Drink ≥ 1 soft drinks a day	%	77.6	75.8	72.9	77.7	
	OR	1.0	0.94	0.83	1.05	
	CI	.	0.7–1.4	0.6–1.1	0.8–1.5	0.17
						
Eat fast food once a week or more	%	48.7	44.5	44.2	50.3	
	OR	1.0	1.18	1.19	0.94	
	CI	.	0.9–1.5	0.9–1.5	0.7–1.2	0.019
						
Fried food for afternoon snack, most days	%	25.7	27.1	26.2	25.5	
	OR	1.0	1.06	1.01	0.98	
	CI	.	0.8–1.5	0.7–1.4	0.7–1.3	0.94
						
Chocolates for afternoon snack, most days	%	30.3	30.5	30.1	30.4	
	OR	1.0	1.04	1.04	1.04	
	CI	.	0.8–1.4	0.8–1.4	0.8–1.4	0.99

^1^P-value for Wald chi-square, controlling for age and gender

Of interest, frequency of family meals was not associated with the home availability of less healthy snack foods (chips, chocolates, and soft drinks) or with the less healthy dietary behaviors (soft drink consumption, fast food consumption, fried foods for afternoon snacks, or chocolates for afternoon snacks) (Table 3). Adolescents who ate family meals everyday were as likely to have less healthy snack foods at home most days and consume them compared with those who did not eat family meals.

## Discussion

The aim of the current study was to examine the relationships between consumption of family meals and BMI, nutrition behaviors and other aspects of the home food environment and that may influence adolescent nutrition. In this large, ethnically diverse sample of adolescents, 42% ate a meal with their family on all of the previous five school nights and younger adolescents were more likely to do so than older adolescents. These findings are consistent with previous research conducted in the US that show similar percentages of young people eating meals with their families most days of the week and a similar relationship with age [5,6,10]. The decrease in eating family meals among older adolescents may reflect the increased autonomy and/or busier schedules of older adolescents as they are more likely to report scheduling difficulties and lack of time for family meals than younger adolescents [16].

Previous research documenting the positive nutritional benefits associated with eating family meals [5-8] has not fully explored possible mechanisms to explain these relationships. We found that adolescents who have family meals perceive more parental support for healthy eating, have limits on television use, and are more likely to have fruits and vegetables available at home every day. A systematic review of the literature identified home availability of fruits/vegetables, frequent family meals, and parental support for healthy eating as potential determinants of fruit and vegetable consumption [17]. Findings from our study also indicate that adolescents who eat family meals are more likely to bring their food for school from home and eat breakfast at home, suggesting that someone in the home is ensuring that there are available foods for adolescents to eat.

An important finding of this research was that there were no significant relationships observed between frequency of family meals and the availability or consumption of high-sugar/high-fat snack foods and fast food. Adolescents who regularly ate family meals were as likely to have less healthy snack foods available at home and regularly eat them as adolescents who do not have family meals. It appears that while families may prioritize eating together, messages about the availability and consumption of these snack foods are not getting through. The high prevalence of the availability of less healthy snack foods in homes may, in part, explain the insignificant relationship between family meals and BMI. Even among adolescents who ate family meals every night in the past school week, more than 40% had chips available at home most days and more than 30% had soft drinks available at home most days. That there was no association between frequency of family meals and BMI may also be explained by other factors not measured in the current study, such as parental education or family work schedules.

Our current study is strengthened by the large, ethnically diverse population of adolescents who participated and the use of objective measures of weight and height. That said, there are several limitations to our study that are important to acknowledge. First, due to the ethnic composition of our sample, extrapolation to the rest of New Zealand or internationally requires additional research. Second, our measure of frequency of family meals only included school days; weekend routines and practices may differ. Previous research has documented that children eat more snack foods, dietary fat, and unhealthy foods on the weekends compared with weekdays [18,19]. Similarly, the current study would have been improved by testing the reliability and validity of the dietary questions during the pilot study. Lastly, there may be other factors that are more important indicators of the family food environment that have not been addressed in this study. Issues like household crowding or parental education or work schedules may mean that young people may be less likely to eat meals with their families and have fewer healthy foods available to eat. It is also possible that the relationship between family meals and BMI may be confounded by physical activity. Our study assessed physical activity over a segmented day to assess specific components of the intervention rather than with a comprehensive measure of physical activity. This means, unfortunately, that controlling for the effects of physical activity was not possible.

## Conclusion

Our findings suggest that there are a number of positive aspects of the home food environment associated with family meals that may be potential mechanisms for the positive associations between family meals and improved adolescent nutrition. Interventions to promote family meals should recognise the issues that prevent families from eating together and acknowledge other aspects of the home food environment promoting healthy eating. Perhaps the most salient implication is that interventions with families need to address the availability and consumption of unhealthy snack foods at home.

## Competing interests

The authors declare that they have no competing interests.

## Authors' contributions

JU conceived the research questions, performed the statistical analysis, and drafted the manuscript. RS and DS acquired the data, participated in the interpretation of the findings, and critically reviewed the manuscript. CNM participated in the interpretation of the findings, and critically reviewed the manuscript. All authors read and approved the final manuscript.
